# Bulk mRNA-sequencing data of the estrogen and androgen responses in the human prostate cancer cell line VCaP

**DOI:** 10.1016/j.dib.2024.111053

**Published:** 2024-10-23

**Authors:** Camille Lafront, Lucas Germain, Étienne Audet-Walsh

**Affiliations:** a Department of Molecular Medicine, Faculty of Medicine, Université Laval, 1050 avenue de la Médecine, Québec City, Québec G1V 0A6, Canada; b Endocrinology – Nephrology Division of Centre de recherche du CHU de Québec – Université Laval, Québec City, Québec, Canada; c Centre de recherche sur le cancer de l'Université Laval, 9 rue McMahon, Québec City, Québec G1R 3S3, Canada

**Keywords:** Steroids, Estrogen receptor, Androgen receptor, SERMs, Tamoxifen, Benign prostate hyperplasia

## Abstract

Prostate cancer is a hormone-dependent disease that relies on the androgen signaling, as well as on the estrogen signaling, for growth and survival. To identify the genes regulated by these sex-steroid hormones in the human prostate cancer cell line VCaP, these cells were treated for 24 h with either androgens and/or estrogens. Then, the RNA of each sample was purified for sequencing to generate bulk mRNA-seq data. After verifying raw quality, reads were pseudo-aligned on the human reference transcriptome (Gencode v27). Analysis was carried out on aligned and quantified data to determine the transcriptomic changes following each hormonal treatment. These data presented herein can be reanalyzed with specific fold-change thresholds for gene expression, or with different pair-wise combinations to compare the hormones’ transcriptional impacts on VCaP cells and better understand prostate cancer cell biology.

Specifications Table

**Table:** 

Subject	Cancer Research.
Specific subject area	Hormonal transcriptomic response (androgenic and estrogenic) in a human prostate cancer cell line*.*
Type of data	Bulk mRNA-seq, Figures Raw, Analyzed, Filtered.
Data collection	After steroid deprivation, VCaP cells that express both the androgen receptor (AR) and the estrogen receptor alpha (ERα) were treated with either estradiol (E_2_; an estrogen) and/or R1881 (a synthetic androgen) in triplicates. Then, 24 h later, RNA was purified from these cells and sequenced to generate bulk mRNA-seq data with an Illumina NovaSeq 6000. Raw data were cleaned and controlled for quality using Trim_Galore, FastQC and MultiQC. Reads were aligned against human reference transcriptome (Gencode v27) using Kallisto. Normalized counts were used in Gene Set Enrichment Analysis tool, and transcriptomic changes were evaluated using the hallmark gene sets collection. All parameters were kept at default.
Data source location	• Institution: Next Generation Sequencing Platform of the CRCHUQ-Université Laval • City/Town/Region: Québec City, QC • Country: Canada
Data accessibility	Repository name: Gene Expression Omnibus Data identification number: GSE256370 Direct URL to data: https://www.ncbi.nlm.nih.gov/geo/query/acc.cgi?acc=GSE256370
Related research article	C. Lafront, L. Germain, G.H. Campolina-Silva, C. Weidmann, L. Berthiaume, H. Hovington, H. Brisson, C. Jobin, L. Frégeau-Proulx, R. Cotau, K. Gonthier, A. Lacouture, P. Caron, C. Ménard, C. Atallah, J. Riopel, É. Latulippe, A. Bergeron, P. Toren, C. Guillemette, M. Pelletier, Y. Fradet, C. Belleannée, F. Pouliot, L. Lacombe, É. Lévesque, É. Audet-Walsh, The estrogen signaling pathway reprograms prostate cancer cell metabolism and supports proliferation and disease progression, J Clin Invest. 134(11):e170809*.*

## 1 Value of the Data

• These bulk mRNA-seq data were generated to study the estrogen response, along with the androgen response, in a human prostate cancer model (the immortalized cell line VCaP).
• These data are useful to identify genes modulated by estrogens and/or androgens in a human prostate cancer model. These treatments activate the transcriptional functions of hormone receptors, either the estrogen receptor alpha (ERα) or the androgen receptor (AR).
• In particular, the estrogen response is seldom studied in the prostate cancer field, and the availability of sequencing data studying this hormonal impact in this context could be useful to other research groups.
• Studies about the estrogen or androgen responses in prostate cancer, or in other types of hormone-dependent cancer for comparison, can benefit from these data.
• These data can be reanalyzed with a precise gene expression threshold notably by selecting precise fold-change cutoffs, or with different pair-wise combinations to compare treatments’ impact on the VCaP transcriptome.

## 2 Background

The estrogen response in prostate cancer (PCa), with or without the co-activation of the androgen response, remains mostly unknown, as recently highlighted by Lafront and colleagues [1]. Indeed, both androgen and estrogen receptors (respectively, AR and ERα) are ligand-activated transcription factors, being members of the nuclear receptor gene family [2]. By treating an immortalized prostate cancer cell line with estrogens and androgens then subsequently sequencing the mRNA, transcriptional modulations induced by distinct hormonal exposures can be studied. VCaP cells were specifically selected for this purpose since they represent a unique *in vitro* model of human prostate cancer, expressing both wild-type AR and ERα [1]; indeed, other commonly used human prostate cancer cell lines are either negative for both receptors, only express AR, or express a mutated AR that can also bind estrogens [3,4]. The bulk mRNA-seq dataset presented herein allows a better understanding of the impact of estrogens as well as androgens in human prostate cancer.

## 3 Data Description

**Fig. 1:**
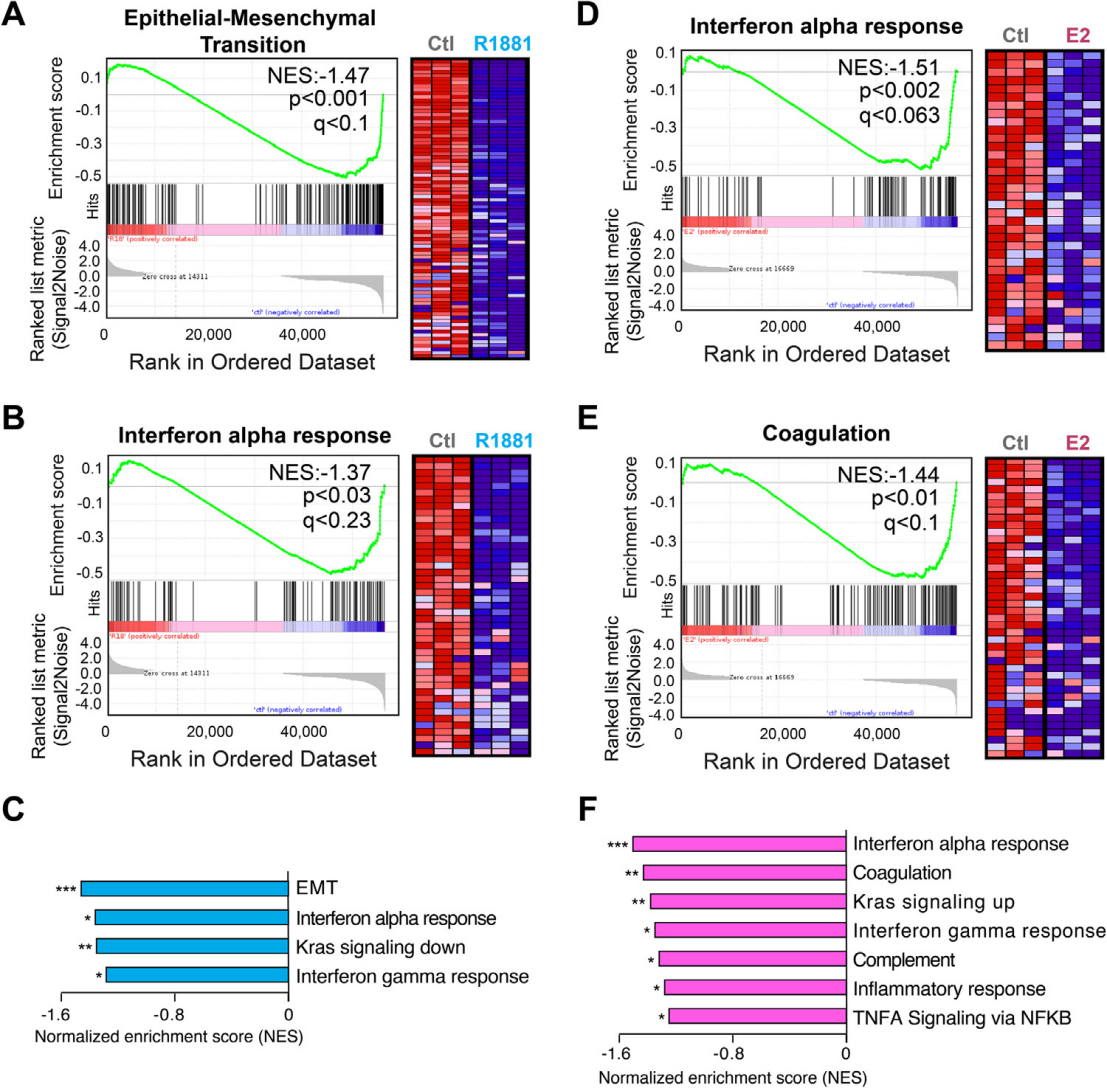
Androgens and estrogens significantly downregulate both similar and distinct biological pathways in the VCaP cell line. (A, B) Gene Set Enrichment Analysis (GSEA) diagrams and heatmaps of the Epithelial-Mesenchymal Transition (EMT) (A) and interferon alpha response (B) gene sets following treatment with R1881 (a synthetic androgen) compared to control (Ctl). (C) Normalized enrichment scores (NES) of all significantly downregulated gene sets following treatment with R1881 compared to Ctl. (D, E) GSEA diagrams and heatmaps of the interferon alpha response (E) and coagulation (F) gene sets following treatment with estradiol (E_2_) compared to Ctl. (F) Normalized enrichment scores (NES) of all significantly downregulated gene sets following treatment with E_2_ compared to Ctl. For the diagrams and heatmaps (A, B, D and E), NES, *p*-values and false discovery rates (FDR, *q*-values) are indicated. Only core genes of each gene set are shown in heatmaps. For NES histograms (C, F), *p < 0.05, **p < 0.01 and ***p < 0.001.

The raw data generated from sequencing are available on the Gene Expression Omnibus (GEO) repository and can be accessed with the reference number GSE256370 [5]. It is composed of 12 samples, precisely of four treatment groups (control, R1881 [a synthetic androgen], estradiol and estradiol + R1881), with three replicates per group. In the related research article, both normalized and raw quantified counts can be found for each replicate in supplementary files in CSV format. The related research article associated with these data focused on the upregulated genes and pathways following sex steroid treatments in VCaP cells [1]; herein, the data were used to evaluate the downregulated transcriptional response following androgen and estrogen stimulation with the Gene Set Enrichment Analysis (GSEA) tool [6] (Fig. 1).

## 4 Experimental Design, Materials and Methods

1. Cell culture and treatments
  The VCaP (vertebrae cancer of the prostate), an androgen receptor (AR) and estrogen receptor alpha (ERα)-positive human PCa cell line [1], was obtained from the ATCC. The cells were re-authentified in 2016 with the ATCC cell line authentication service, which uses an assay that identifies short tandem repeat markers specific to every *in vitro* model established (FTA Sample Collection Kit for Human Cell Authentication Service [ATCC 135-XV]). VCaP were kept in culture for less than four months after resuscitation and were frequently tested for mycoplasma contamination. They grew in DMEM media supplemented with 10 % fetal bovine serum (FBS) and 1 % streptomycin, penicillin, and sodium pyruvate, and were incubated at 37 °C with 5 % CO_2_. The media was changed two to three times per week, and cell confluence was kept below 70 %.
  Two days before treatments, to ensure steroid deprivation, the cells were seeded in 6-well plates (0.5 M cells/well) with RPMI-1640 media with no phenol-red supplemented with 5 % charcoal-stripped serum (CSS), and 1 % streptomycin, penicillin and sodium pyruvate. Then, media were renewed, and the cells were treated with either vehicle (ethanol 96 %; Sigma), 17β-estradiol (E_2_; 10 nM; Sigma; agonist of ERα), R1881 (metribolone; 10 nM; Toronto Research Chemicals; agonist of AR) or a combination of both. Each treatment was done in triplicates, resulting in a total of 12 samples. A day later, media were removed and cells were frozen at −20 °C until RNA extraction.
2. RNA extraction and sequencing
  Total RNA of treated VCaP cells was extracted with the RNA purification kit RNeasy Mini Kit from QIAGEN by following the protocol given by the company, and was kept at −80 °C until sequencing. Excellent RNA integrity was confirmed using a TapeStation 2200 (Agilent), where all samples had an equivalent RNA integrity number (RINe) > 8. mRNA enrichment and library preparation were performed using the NEBNext Ultra II Directional RNA library prep kit following the manufacturer's protocol. RNA was then sent to sequencing at the Next Generation Sequencing Platform of the CRCHUQ-Université Laval (based in Québec City) using an Illumina NovaSeq 6000 (paired-end, 100 bp sequence length, depth of 20–25 M reads requested).
3. Bulk mRNA-seq data analysis
  After sequencing, raw data were obtained in the fastq format. Depth ranged from 25 to 32 M reads before trimming, and from 24 to 31 M after trimming. Data quality control was performed using FastQC and MultiQC [7,8], before and after the data were cleaned of adaptor content and low-quality reads using Trim_Galore/Cutadapt on default parameters [9,10]. The Kallisto tool was used for the pseudo-alignment of the trimmed sequences to the Gencode v27 human reference transcriptome, also on default parameters [11]. Finally, to study pathways downregulated by sex steroids in VCaP cells, the GSEA tool was employed [6], permutating gene set a 1000 times and using Broad Institute's hallmark gene sets collection. Other parameters were kept at default.

## Limitations

None.

## Ethics Statement

The authors declare to have followed the ethical requirements for publication in Data in Brief and confirm that the work presented herein does not involve human subjects, animal experiments or any data collected from social media platforms.

## CRediT Author Statement

**Camille Lafront:** Conceptualization, Methodology, Validation, Formal analysis, Investigation, Writing – Original Draft, Review & Editing, Visualization, **Lucas Germain:** Software, Validation, Formal analysis, Investigation, Data curation, **Étienne Audet-Walsh:** Conceptualization, Formal analysis, Writing – Original Draft, Review & Editing, Visualization, Supervision, Project administration, Funding acquisition.

## Data Availability

GEO)The estrogen and androgen responses in the immortalized human prostate cancer cell line VCaP (Reference data).
